# Digital linguistic sovereignty and Kazakh language policy in the digital environment

**DOI:** 10.3389/fpsyg.2026.1858650

**Published:** 2026-09-03

**Authors:** Sholpan Zharkynbekova, Gulbagira Ayupova, Bakhyt Galiyeva, Zukhra Shakhputova, Anastassia Zabrodskaja

**Affiliations:** 1 Department of Theoretical and Applied Linguistics, L.N. Gumilyov Eurasian National University, Astana, Kazakhstan; 2 Department of Foreign Languages, L.N. Gumilyov Eurasian National University, Astana, Kazakhstan; 3 Baltic Film, Media and Arts School of Tallinn University, Tallinn, Estonia

**Keywords:** access to digital ecosystems, digital language policy, Kazakhstan, linguistic sovereignty, national digital infrastructure, the Kazakh language

## Abstract

This article examines the impact of the digital environment on the status and place of language. The focus is on the concept of digital linguistic sovereignty, understood as a nation’s ability to ensure the functional competitiveness of the language in the age of artificial intelligence and big data. In this regard, Kazakhstan presents a unique case study of a country transitioning from traditional language protection to the active construction of digital sovereignty. As a state undergoing active nation-building, the country simultaneously demonstrates one of the highest levels of digitalisation in the post-Soviet space. The implementation of large-scale state programmes (such as “Digital Kazakhstan”) has led to the formation of a new communicative environment – a virtual linguistic landscape – in which the dynamics of interaction between the state language and global languages are assuming qualitatively different forms. The methodological basis of the study is an interdisciplinary approach combining critical discourse analysis of official documents, institutional analysis of technological projects such as the National Corpus of the Kazakh Language, the Coursera platform in Kazakh, the translation of educational materials for higher education institutions, and others, as well as the study of expert discourses in new media. The study’s findings illustrate how the state regulates the process of language planning in the context of increasingly pervasive technological advancement, transitioning from a protective model toward digital modernisation. The creation of mass digital content in academic and technical fields has been demonstrated to contribute to the growing intellectual recognition of the Kazakh language. The authors of the study argue for the need to transition from formal status planning to an inclusive model of digital citizenship in order to achieve “linguistic justice.” It has been established that language digitalization is becoming a tool of “soft power,” transforming civic identity and expanding access to digital capital. The practical significance of this study lies in the potential application of the proposed analytical framework to the study of language processes in other multilingual societies undergoing digital transformation.

## Introduction

The current phase of societal development is distinguished by substantial transformations that are inextricably linked to the influence of digital technologies, thereby posing novel and fundamental challenges to the scientific community. However, rapid digitalization is not a socially neutral process. In recent years, scholarly discourse has increasingly voiced the idea that the digital environment can not only promote inclusion but also generate new forms of social and linguistic inequality. For instance, Kornai demonstrated that fewer than 5% of the approximately 7,000 languages in existence today have the capacity to achieve a full digital presence, while the remainder are on the brink of “digital extinction” – a process that largely does not coincide with traditional indicators of offline linguistic vitality (Kornai, 2013). At the institutional level, similar concerns are reflected in the European Parliament’s resolution on linguistic equality in the digital age, which documented a pronounced imbalance in the technological support of European languages and proposed measures to overcome it (European Parliament, 2018). Using Netherlands as a case study, Smit et al. (2025) demonstrate that digital citizenship is experienced differently by groups of citizens with limited communicative and digital capabilities, challenging the universalistic assumptions underlying many digital inclusion policies. In this context, the concept of digital citizenship is inextricably linked with issues of linguistic justice. This concerns citizens’ right to full communication, access to information and participation in the life of the digital state without discrimination on the basis of language. In this regard, language policy, traditionally understood as a set of ideologies, practices and institutions aimed at regulating the linguistic situation (Spolsky, 2004) faces the need to expand its analytical framework to take into account the conditions in which language exists and functions in the digital environment. Electronic media resources and online platforms create spaces that are, in many respects, analogous to the language markets and fields as conceived by Bourdieu, 1991, within which linguistic practices and norms of linguistic visibility take shape. Kelly-Holmes (2023) demonstrates that, under conditions of algorithmic governance, these norms are increasingly shaped not by institutions in the traditional sense, but by automated systems that reproduce users’ established linguistic preferences. This shifts a significant portion of linguistic functioning to the digital environment and necessitates an expansion of the theoretical foundations of language policy. As a result, the research focus is on examining language policy in the context of digital transformation, in which the object of analysis is not only language as a system, but also the conditions of its functioning in the digital environment. The interrelationship between these processes lies in the fact that digital transformation, as an institutional process, determines which digital environments are created and maintained by the state, whilst these environments themselves define the specific conditions in which language is used – which languages are supported by the interfaces, recognized by the algorithms, or available via machine translation. In this sense, the implementation of language policy is not solely determined by decisions regarding the linguistic status, but also by technical configurations that enable or impede certain linguistic practices. As contemporary research demonstrates, language in the digital age functions within technological ecosystems that possess their own logic of selection, hierarchy and visibility (Androutsopoulos, 2013, 2014). At the intersection of these strands of research, the concept of digital language policy is gradually taking shape within academic discourse. In this regard, language policy is implemented not only through legislation and educational institutions, but also through technological solutions such as interfaces, algorithms and platform standards which determine the conditions for language choice and its use in the digital environment (Rehm et al., 2020; Kelly-Holmes, 2023). Concurrently, official institutional texts published in the digital environment – for example, university missions on official websites – themselves become a platform on which national and linguistic identity is constructed (Chernyavskaya and Zharkynbekova, 2019). In contradistinction to implicit, bottom-up processes of linguistic convergence, such as koineization, regulation of this kind emanates from state and technical actors – namely platform developers, standardization organizations, and state digital agencies – who determine the technical conditions for language choice, even if these decisions are not always formalized as explicit legal norms.

This distinction is consistent with the classical differentiation between explicit and implicit, as well as overt and covert language policy (Spolsky, 2004; Schiffman, 1996). Explicit policy is typically associated with regulatory decisions made from the top down, for example legislation, government programmes and institutional standards. In contrast, implicit policy is formed from the bottom up through stable linguistic practices that emerge as a result of social processes and are not always normatively enshrined. The present study focuses on explicit institutional language policy implemented through government policy documents and digital infrastructure. Implicit, bottom-up digital linguistic practices, however, remain outside the scope of this paper and are designated as an avenue for further research.

Digital language policy as a phenomenon is closely linked to another concept gradually entering scholarly circulation: “digital linguistic sovereignty” (or “digital autonomy,” “freedom of language”), which is defined, on the one hand, as the ability of a language to exist and develop unhindered in the digital environment, and, on the other hand, as the ability of a state or political community to ensure the autonomous and sustainable development of a language in key digital spheres (Zaugg et al., 2022; Migge and Schneider, 2025). This concept is not confined to content creation; it also encompasses the management of digital infrastructure, the development of machine translation and automatic language processing technologies, the formation of national corpora as reference data sets, and data management policies employed in artificial intelligence systems.

Research on the post-Soviet space demonstrates that digital transformation can simultaneously reproduce historical linguistic asymmetries and create new opportunities for linguistic activism and institutional intervention (Pavlenko, 2008; Suleimenova, 2020). The parallel development of digital transformation processes in the state and language policy is shaping each country’s own model of the conditions for the functioning of languages.

In this regard, Kazakhstan’s experience is of particular interest for scientific research. Despite the growing body of research devoted to specific aspects of the linguistic situation or digitalisation in Kazakhstan, a comprehensive theoretical analysis of the interrelationship between these two large-scale processes remains underdeveloped. However, there is a paucity of attention paid to the manner in which digital infrastructure (codes, interfaces, algorithms) itself becomes a tool of ideology and changes citizens’ everyday practices. To date, there is no clear understanding of how “digital Kazakh” is reshaping the social contract between the state and society. Existing works tend to focus on specific programmes or individual research questions (Sarsenbieva et al., 2021; Serikbaeva, 2023; Serikbaeva and Olach, 2025; Alkebaeva and Sultan, 2025). However, a sociolinguistic analysis based on a comprehensive study of the impact of digital transformations on language planning requires greater attention from specialists. The necessity for a more comprehensive approach to this issue has rendered the present study pertinent.

This article aims to analyze how Kazakhstan is constructing its digital linguistic sovereignty through the creation of a national knowledge infrastructure, and how this infrastructural project is discursively linked to the broader question of maintaining the home language. In line with this goal, the study is guided by two research questions:

(1) How is the concept of “digital linguistic sovereignty” manifested and asserted in state policy documents and institutional strategies for digital transformation in Kazakhstan?
(2) How do the architectural features and affordances of national digital infrastructures (e.g., e-government platforms, national language corpora, and automated translation tools) shape the functional visibility and algorithmic representation of the Kazakh language?

The study contributes to the development of language planning theory by integrating digital studies and offers an analytical framework for studying similar processes in other multilingual societies.

## Literature review

### Digital sovereignty and language infrastructure

Classic typologies of language planning (Spolsky, 2004) are predicated on the “status–corpus–acquisition” framework, regarding the language’s functional environment as a neutral space. The emergence of the digital environment has contributed to a shift in this approach. In this instance, technology does not merely define the norm but shapes it. Algorithmic ranking, interface design, and data processing protocols are de facto becoming instruments of language policy, and in this sense, they provide an important context for the analysis presented in this article. This shifts the focus of contemporary analysis from normative regulation to the governance of digital infrastructure and allows for a rethinking of linguistic sovereignty not as the protection of state borders, but as control over the digital tools of language reproduction. For instance, in the absence of representation of a given language within the algorithms and digital interfaces employed within a national digital space, there exists a possibility that the language may be susceptible to displacement by other languages that are technically more proficient in the same digital systems.

These processes assume particular significance in multilingual states: digitalization can both exacerbate competition between languages (Nelde, 1987) and create conditions for their complementary functional distribution across niches, without necessarily implying rivalry (Mühlhäusler, 1996). In any case, digitalization raises the question of the functional sustainability of language, shifting the emphasis from the symbolic recognition of language to its technological viability.

Within the context of this trend, the concept of “digital sovereignty” assumes paramount significance. This phenomenon occupies a central place in the discussion about whether digitalisation can serve as a tool for supporting linguistic diversity, creating new opportunities for languages to produce and distribute content (Cunliffe, 2007), or whether it entrenches a new form of neocolonial linguistic inequality under the guise of neutral technologies, reproducing the structural dependence of peripheral languages on a technological infrastructure controlled by a limited number of global corporations (Kwet, 2019).

According to Floridi (2020), digital sovereignty is defined as the ability to influence digital elements, including data, software and standards, and their dynamics. With regard to language, this signifies that genuine language independence in the contemporary era is equivalent to control over the digital ecosystem of its processing.

Concurrently, the notion of digital citizenship is undergoing a transformation. Mossberger et al. (2008) define digital citizenship as the ability to participate fully in social life online, highlighting economic opportunity, democratic participation, and inclusion in dominant forms of communication as its key dimensions. This paper expands on this concept by adding a linguistic element: in our opinion, full digital citizenship also implies the right to communicate and access digital services in one’s native or preferred language, as the lack of language support in digital infrastructure limits participation in the same way as the lack of technical access or skills, which is the focus of the original concept.

Building upon this notion, recent studies have introduced the concept of “digital human capital” – a set of digital knowledge and skills that enhance an individual’s capacity to utilize digital technologies effectively (Mossberger and Tolbert, 2021). When applied to the linguistic sphere, this capital takes on the character of a positive externality: state investments in the creation of Kazakh-language digital infrastructure – language corpora, machine translation systems, localized interfaces – once implemented, benefit not only the users for whom this infrastructure was originally created, but also the entire language-speaking community as a whole, since such resources can be used by an unlimited number of people without additional marginal costs.

Couldry and Mejias (2022) have made a significant contribution to the understanding of these mechanisms by proposing the concept of “data colonialism” to describe the appropriation of human lives through data. The authors demonstrate that data is extracted from everyday life and accumulated by corporate capital, forming a new global regime of control. This consistent pattern is replicated in the linguistic sphere, giving rise to a structural process of digital linguistic colonialism. The phenomenon can be characterized as follows: data from speakers of minority languages is used to train models that subsequently fail to ensure functional parity for these languages. According to Kornai (2013), approximately 98% of the world’s living languages are not supported on the most popular devices and platforms, and no more than 5% of languages have the potential to achieve digital vitality. The scholar warns that the remaining 95% could face “digital extinction” (Kornai, 2013). Some languages are struggling to survive only due to a critical shortage of machine-readable resources and state support (Kirchmeier, 2020). These facts support the assumption that state attempts to ensure linguistic sovereignty may collide with a technological reality where platforms own communication channels and attention-distribution algorithms.

In response to the issues identified, Zaugg et al. (2022) introduce the concept of “digitally disadvantaged languages.” These are defined as languages facing multiple inequalities in the digital environment, including gaps in digital support and vulnerabilities to digital surveillance. Building upon this logic, Keet and Khumalo (2023) proposed a methodology for assessing this status using a five-level “resourcefulness” matrix. In contradistinction to technocratic approaches, the model under scrutiny incorporates eleven dimensions, encompassing both technical aspects (e.g., corpora, tools) and sociolinguistic factors (e.g., policy, funding). The authors thus posit that the degree of resilience exhibited by a given language is contingent upon the intricate interplay of institutional and technological factors. However, despite the existence of these frameworks, it remains unclear what specific combination of resources – technical infrastructure (encoding, font support, input systems), the volume and quality of language data, institutional and financial support from the state, as well as specialized, regionally adapted resources that take into account the cultural and legal context of a particular country – is necessary and sufficient for a sustainable digital presence of a language. The extant research demonstrates that the presence of a single element does not guarantee a complete result: a translated interface alone does not eliminate algorithmic asymmetry (Mpofu et al., 2023), and the participation of a language in multilingual models does not provide comparable security without specialized regional data (Goloburda et al., 2025). Digital linguistic sovereignty is therefore understood in this article not as the possession of a single resource, but as a state’s capacity to build and simultaneously maintain the entire necessary set of conditions – technical, institutional and substantive – which determine the real autonomy of a language in the digital environment.

Research indicates that modern language technologies are not neutral, as they reproduce structural inequalities by reinforcing the dominance of English-language content and displacing low-resource languages (Bender et al., 2021). Furthermore, training models on open internet data reinforces existing stereotypes, leading to increased algorithmic bias. For instance, Mohr’s (2022) study reveals that in Zanzibar’s digital tourism landscape, English predominates as a means of visibility (93.8% of content), while local languages (particularly Kiswahili) are employed symbolically to exoticise the space. This suggests that digital platforms have the potential to perpetuate linguistic colonialism, whereby local languages are devoid of functionality and become markers of authenticity for consumption by a global audience. Consequently, the absence of explicit support standards becomes not just a technical problem, but a factor of increasing discrimination (Kuulmets et al., 2024; Togmanov et al., 2025).

However, technological development is not the only prospect. The literature on language revitalisation has demonstrated that an increase in a language’s public visibility and prestige is one of the key conditions for its preservation. Thus, Cooper (1989) classifies prestige planning as one of the independent strands of language planning, alongside status planning, corpus planning and language acquisition planning. Fishman (1991) links the resumption of intergenerational language transmission with the expansion of its spheres of use and an increase in status. Grenoble and Whaley (2006) attribute the increase in language prestige to the factors determining the success of revitalization programs. Today, the digital environment serves as a platform on which these approaches are implemented simultaneously: expanding a language’s digital presence functions as prestige planning, as corpus planning – through the creation of language resources and terminology – and as language acquisition planning – through digital educational platforms. In this sense, digital linguistic sovereignty can be viewed as an institutional framework that brings together traditional areas of language planning as applied to the digital environment. Empirical evidence for this hypothesis can be found in Eke and Salawu’s (2026) study, which demonstrates that the Igbo language’s sustained digital presence on the BBC News Igbo platform has contributed to the restoration of its prestige, interest, and expansion of its use. Tripathi (2025) explores parallel challenges to linguistic survival in the digital environment, using India as a case study in another multilingual context. This research demonstrates that a similar dynamic – the dependence of language viability on its digital presence and institutional support – can be observed outside the African context (Tripathi, 2025).

Nevertheless, the condition of institutional support identified by Eke and Salawu (2026) is implemented differently across various regions. In the context of the post-Soviet space, particularly Central Asia, this necessitates a consideration of the specific institutional trajectory, wherein the mechanisms for translating global processes are mediated by the legacy of Soviet language policy and contemporary digitalisation strategies. In contradistinction to Western models, in which development is frequently market-driven, in this region the historically established interconnection between a language’s official status and its digital presence plays a pivotal role. Neglecting this factor may lead to an inaccurate interpretation of a language’s “resourcefulness,” given that formal support does not always correspond with actual digital activity.

### The digital language landscape in the post-soviet space: current status and challenges

Research into language policy in post-Soviet states demonstrates the intricacy of the shift from the Soviet model to distinct national strategies. Even today, the policies of independent republics continue to be viewed through the prism of decolonisation, where the language of the titular nation is granted official status to strengthen national sovereignty.

A comparative analysis of the Estonian experience reveals how the characteristics of the digital linguistic ecology and institutional regulation vary depending on the historical legacy of a particular society. As demonstrated by Fedorova and Tšuikina (2024) in the context of Tallinn, even in a highly digitalised state, complex patterns of language interaction persist in public spaces. This lends support to the argument that technological sophistication alone does not resolve issues of linguistic inclusion (Fedorova and Tšuikina, 2024). The Estonian experience demonstrates that the country is among the world leaders in the Digital Government Index (OECD, 2025). From a linguistic measurement perspective, this digital maturity is supported by targeted public policy. Since 2006, Estonia has consistently implemented public funding programmes for language technologies. The present stage is characterized by the strategy “Estonian Language Technology 2018–2027,” (Ministry of Education and Research of the Republic of Estonia, 2018) which is focused on the creation and updating of language resources, the development of speech technologies, and the creation of linguistic tools (OECD, 2026).

According to the 2021 census data cited by the minority rights monitoring body, 29 per cent of Estonia’s population speak Russian as their mother tongue (SHR Monitor, 2026), yet digital public services do not always take the linguistic needs of this group into account. The 2024 Nordregio report notes that “immigrants who do not speak the official language” are identified as one of seven target groups at risk of digital exclusion and require special measures to ensure the accessibility of services (Nordregio, 2024).

As Petrovica et al. (2022) observes, although Estonia exhibits elevated levels of digitalisation as a result of strategies such as “Tiger Leap,” empirical data highlights a persistent discrepancy between the normative linguistic landscape and the actual patterns of language use. While Estonian dominates signage and interfaces visually due to state language legislation requirements, Russian predominates in the oral communication of the Russian-speaking minority. According to the Statistics Department of Estonia, at the beginning of 2026, ethnic Russians constituted 276,125 people (approximately 20.3% of the total population), while the Russian-speaking population as a whole reached approximately 29% (Statistics Estonia, 2026). This phenomenon is especially evident in areas where this minority population is concentrated, such as Narva. This contradiction demonstrates that the formal incorporation of language in interfaces does not intrinsically ensure linguistic justice for a linguistic community whose quotidian oral practices deviate from those prevalent in public and digital domains. According to human rights monitoring, this deviation engenders risks of social exclusion and has the potential to exert destabilizing influences on the broader society (SHR Monitor, 2026).

It is evident that the digital landscape of the online space is emerging as a novel arena in which the functioning of multilingualism necessitates in-depth analysis and the development of new, effective projects. For instance, contemporary studies of the post-Soviet space underscore the intricacy of the transition from the Soviet model of language planning to national strategies (Pavlenko, 2008; Smagulova, 2008). Consequently, Soviet language policy, typified by mass Russification, resulted in an uneven yet stable linguistic shift among the ethnic Kazakh population. According to the 1989 census, ethnic Kazakhs constituted only 40.2% of the republic’s population (Results of the Population Census, 1990). A significant portion of urban ethnic Kazakhs adopted Russian as their primary means of communication (Dave, 2007). Surveys of the urban population in the 1980’s and 1990’s recorded an extremely low level of fluency in Kazakh among this group (Dave, 2007). Decades of Russian language dominance resulted in a persistent imbalance in Kazakh-Russian bilingualism, with Russian predominating even among native Kazakh speakers. This phenomenon was evidenced by both the functional distribution of the two languages and a decline in Kazakh proficiency among L1 speakers (Smagulova, 2008). This linguistic shift was distributed geographically unevenly, exhibiting variation between northern regions characterized by predominantly Slavic populations and southern regions with a higher concentration of ethnic Kazakhs (Fierman, 2006).

Today, the situation has changed. However, multilingualism, the continued stability of Russian as a lingua franca, and the spread of English as a mandatory tool for global communication and technology (Liddicoat and Kirkpatrick, 2020; Yedgina et al., 2023) create certain challenges that manifest themselves in practice. A notable illustration of this phenomenon can be observed in the study conducted by Kucherbayeva and Smagulova (2026), which disclosed a considerable incongruity between the formal language policy and the prevailing circumstances in educational settings. It has been demonstrated that, despite the prevailing assumption that Kazakh-language schools provide monolingual instruction, in practice, teachers frequently resort to using Russian and English to facilitate students’ understanding of the material and overcome difficulties associated with their inadequate Kazakh language proficiency and limited educational resources (Kucherbayeva and Smagulova, 2026).

Digital platforms are becoming spaces where users become active and influential participants in shaping language policy from below, constructing their own identities, and circumventing the restrictions imposed by traditional gatekeepers (Bowe et al., 2012, p. 14). For instance, the Kazakhstani platform *Janasozdik* functions as an effective instrument for “Kazakhization from below,” utilizing crowdsourcing mechanisms to engage mass users in solving complex problems (Eisenberg, 2021). The theoretical concepts outlined above find concrete expression at the level of individual digital platforms, which are analyzed further in this article. Thus, a platform’s affordances are evident in the languages supported by the interface and search engine of the National Corpus of the Kazakh Language and the eGov.kz portal. The logic of the language market (Bourdieu, 1991) can be traced in the way Kazakh-language and Russian-language content compete for visibility and legitimacy on the Kazakh-language Wikipedia. Networked multilingualism, as understood by Androutsopoulos (2014), is evident in the way users of the Coursera platform choose their language of interaction depending on their intended audience and the communicative situation. A detailed analysis of how these mechanisms operate on each platform is presented in the Findings section.

It is important to note that the effectiveness of a digital linguistic presence is determined not only by the volume of content, but also by its infrastructural accessibility. Research by the UNESCO Office in Almaty (2021) demonstrated that in Central Asian countries, mobile technologies have become a significant means of facilitating access to education for populations lacking stable internet access. This experience highlights that digital linguistic presence strategies require adaptation to local infrastructural realities, including bridging the digital divide in remote regions (UNESCO Office in Almaty, 2021).

Thus, this review suggests that, at the intersection of diverse studies of language policy in the context of global challenges, the creation of digital infrastructure for low-resource languages in automated systems remains understudied. The study addresses this lacuna by analyzing how strategic steps taken by Kazakhstan (the development of new digital platforms, translation practices, and the localization of text content) are ensuring the transition from institutional declarations to the actual presence of the Kazakh language in the global digital space and shaping new standards for language use in automated systems.

## Materials and methods

This study analyzes explicit language policy, i.e., the officially recorded, declared, and implemented norms, decisions, and practices in the field of language by the state and its institutions. The methodological framework of the study is based on the principles of an interdisciplinary approach, incorporating content analysis and critical discourse analysis. The unit of analysis is the “digital language policy” complex, understood as the combination of official discourses, institutional practices and technological tools aimed at regulating linguistic existence in the digital environment. The authors examine explicit declarations concerning the status of the Kazakh and Russian languages in legislation, strategies and programmes, and the discursive strategies employed to legitimize linguistic decisions.

A triangulation of methods was employed to collect and analyze the empirical data, thereby ensuring the validity and completeness of the findings. To conduct a qualitative content analysis and identify words in the “Digitalisation” thematic cluster, a quantitative analysis of word frequency was carried out in the texts of three state programmes –State Programme for the Development and Functioning of Languages in the Republic of Kazakhstan for 2011–2019 (2011), the State Programme for the Implementation of Language Policy in the Republic of Kazakhstan for 2020–2025 (2019), the Concept for the Development of Language Policy in the Republic of Kazakhstan for 2023–2029 (2023). The study included official Russian-language versions of government documents posted on official websites.^1^,^2^ Since official translations of documents into Russian are performed by authorized bodies, undergo a two-stage linguistic and legal review as part of the legislative process, and have equal legal force with the Kazakh-language version of the document, we considered the Russian-language versions of the documents to be completely identical to the originals.

The data processing for frequency analysis was conducted using the AntConc software (version 4.3.1). The corpus analyzed comprised 37,325 words, of which the text corpus for the 2011–2019 programme comprised 4,768 units, the 2020–2025 programme – 11,804 items, and the 2023–2029 concepts – 20,753 items. Only lexically independent parts of speech with a nominal meaning were included in the analyzed corpus; function words were excluded using a specially created stop list. The stop list was generated within the same Antconc programme using the relevant function. The list of stop words included function words [prepositions, conjunctions, particles, and hybrid words such as “as a result,” “in general,” and “in particular” (*N* = 35)]. The final stop list was approved collectively by the authors of the manuscript.

The final list of full-form words comprised 2,230 items. To identify the keywords for the “Digitalisation” lexical-semantic group, the resulting list was analyzed manually, a decision justified by its modest size and the need for accurate interpretation of the words. To minimize the impact of homonymy and polysemy, contextual filtering and manual verification were applied: the meanings of homonyms and polysemous words were first analyzed within a context of ±5 words surrounding the key occurrence, and then checked manually by two researchers. The manual categorisation of keywords was carried out by two researcher-coders, who were native speakers and specialists in the Russian language with academic qualifications and experience in discourse analysis, and was supervised by the academic supervisor of the project within which this study was conducted. No discrepancies in the categorisation of keywords were recorded.

Search patterns (wildcards) were used to account for the morphological variability of the Russian language. Patterns such as lemmatization and suffixal extensions (e.g., yazyk*, yazykov*) were employed. This approach enabled the consolidation of various word forms into key concepts of digitalization. In some cases, cognate nouns and adjectives were used as a single marker word, for example “innovation,” “innovative.”

The frequency was determined by the number of times each word was used in each document. The obtained data were then ranked in descending order. The analysis included words with a density of ≤4. The threshold of four occurrences was determined on the basis of an analysis of the corpus frequency distribution and the principles of corpus discourse analysis. According to Baker (2006), recurrent connections between words provide the strongest evidence of the existence of discourse.

The threshold of four occurrences ensures the minimum frequency required to identify stable discursive patterns, whilst simultaneously excluding statistically unstable units falling within the “long tail” of Zipf’s (1949) law. The frequency analysis enabled us to obtain quantitative data for further qualitative interpretation (identifying discursive patterns, strategies, ideological mechanisms, etc.) and ensured the methodological validity of the study. At the level of discursive practice, emphasis was placed on raising issues pertinent to the questions discussed in the article, constructing a narrative about digitalization, and identifying the connections between emergent issues. This methodol an empirical case study, thereby unveiling the und ogical approach enabled the transformation of public discourse from a source of information into erlying ideological mechanisms that shape Kazakhstan’s digital language policy.

Critical discourse analysis (CDA) is based on Fairclough’s (1992) three-dimensional model, which links textual analysis, discursive practice, and social practice, as well as van Dijk’s (1993) approach to identifying ideological positions in discourse through the analysis of nomination, modality, and legitimation strategies. The general methodological framework of critical discourse analysis applied to institutional and political texts is outlined in Wodak and Meyer (2015).

**FIGURE 1:**
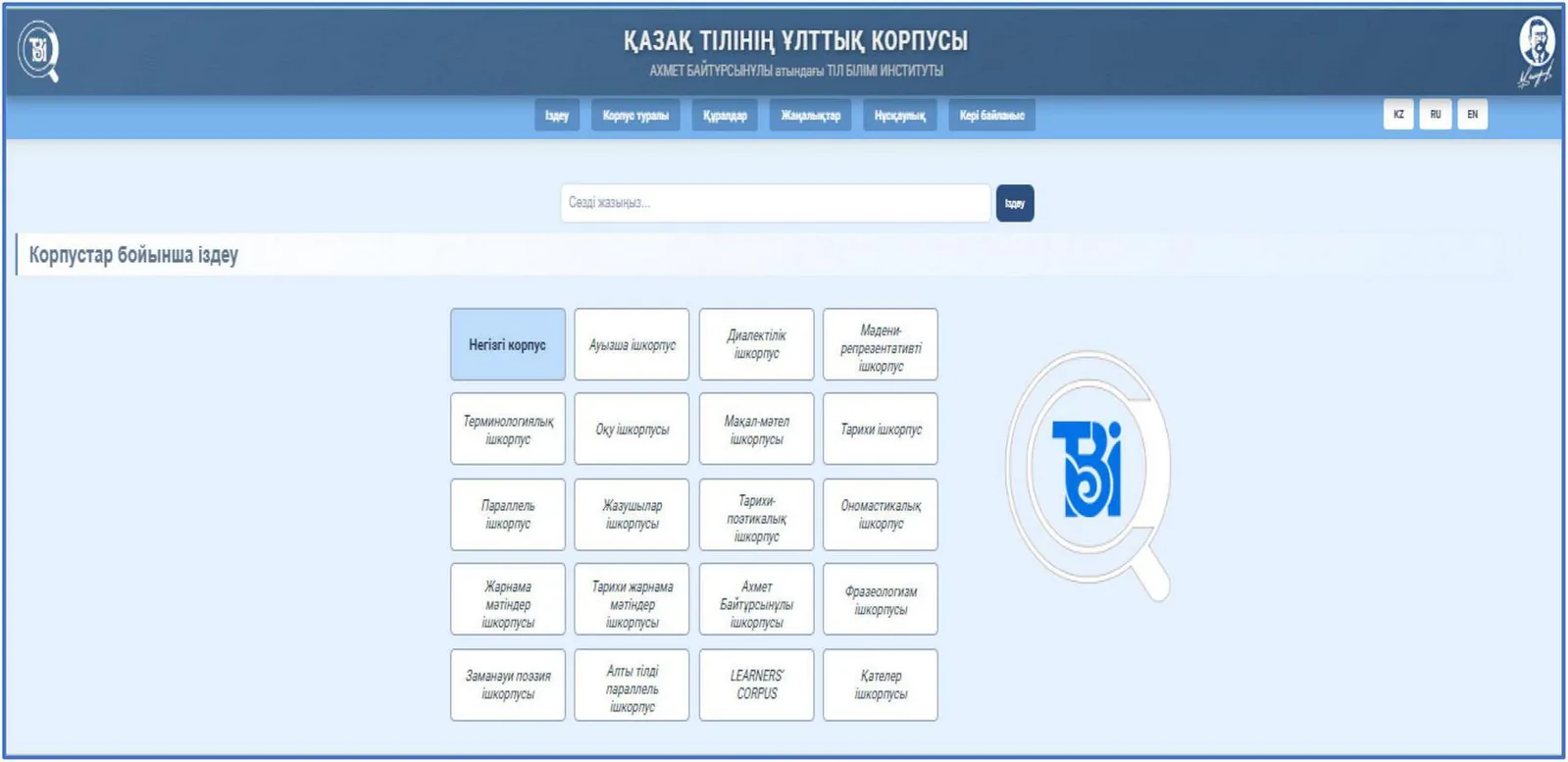
National corpus of the Kazakh language.

**FIGURE 2:**
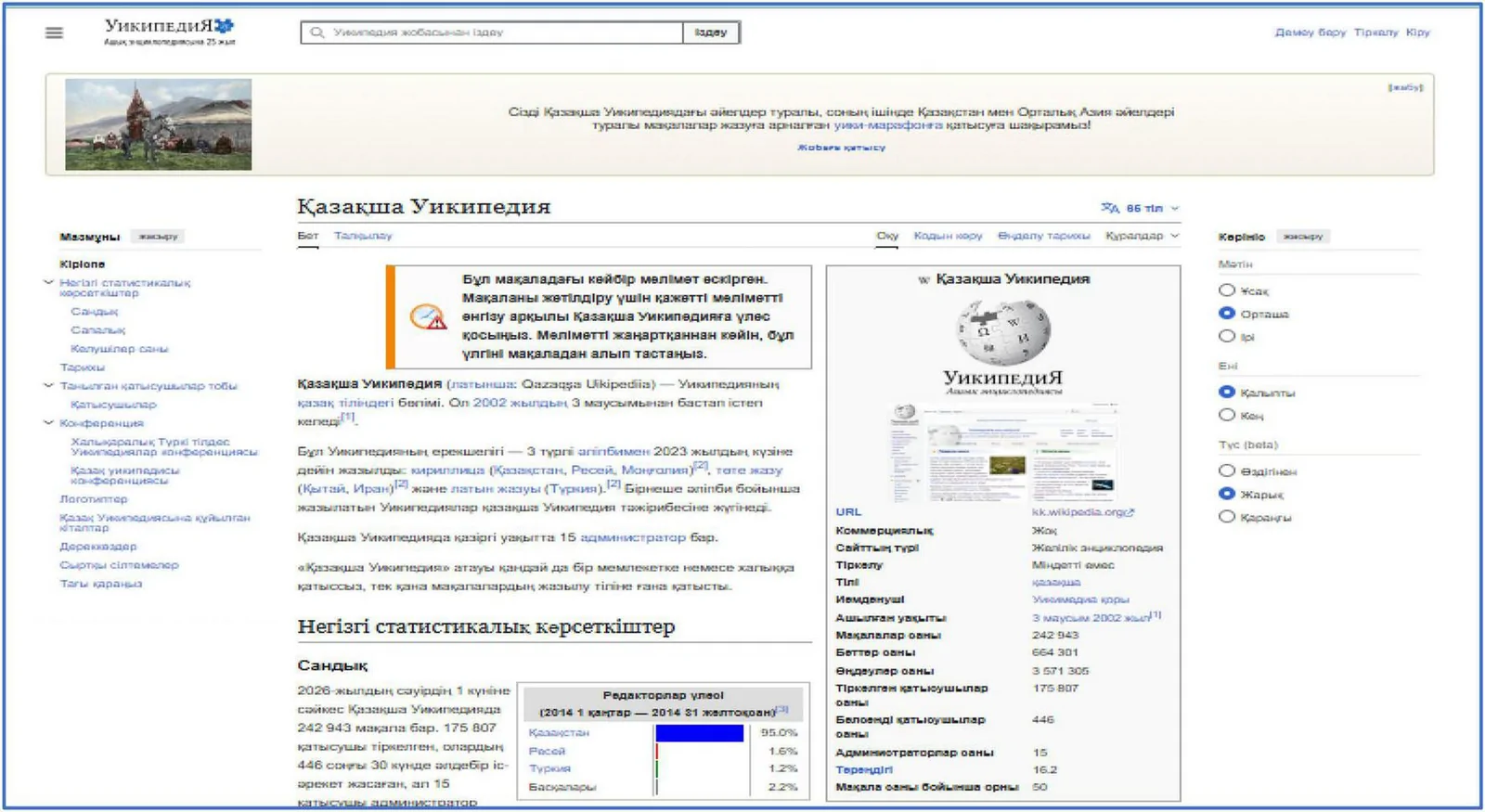
Qazakqsha Wikipedia – the Kazakh-language Wikipedia.

**FIGURE 3:**
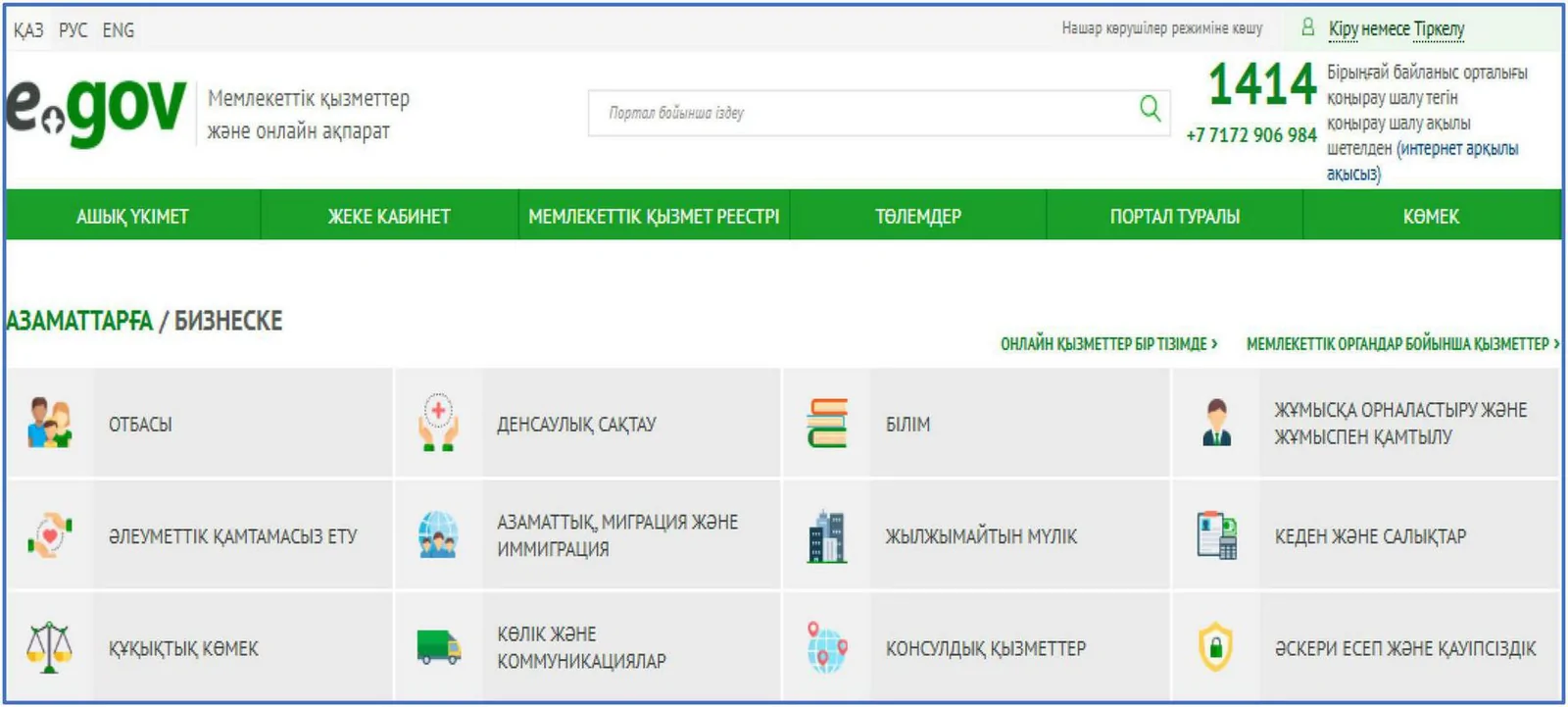
Portal of electronic services of the Government of the Republic of Kazakhstan.

The CDA of official documents enabled us to analyze a set of texts that form the ideological and programmatic basis of Kazakhstan’s language and digital policy for the period 2010–2025. Within the CDA framework, analytical categories such as framing, metaphors, narratives, and ideological mechanisms were employed. The analysis of thematic and lexical-syntactic patterns in the texts resulted in the identification of problematic definitions, cause-and-effect attributions, moral evaluations, and recommendations. The analysis of metaphorical expressions facilitated the identification of conceptual metaphors, while the examination of actantial patterns and elements of the texts’ macrostructure enabled the identification of narratives. The analysis of modality and argumentative algorithms in the texts enabled the drawing of conclusions about the ideological mechanisms employed. The platforms (Figures 1–3) were selected based on the principle of sufficient diversity, covering four key functional domains of Kazakhstan’s digital space (Table 1):

**TABLE 1:** Justification for the selection of digital domains of Kazakhstan for the critical discourse analysis (CDA).

Platform	Domain	Reasons for selection
eGov.kz (https://egov.kz/cms/kk)	Public services (e-government)	The largest government portal, a barometer of language policy in the field of public services
Coursera (www.coursera.org/kk)	Educational domain (EdTech)	A flagship project for the digitalization of education, reflecting the status of language in academic discourse
Wikipedia (https://egov.kz/cms/kk)	Crowdsourcing/information domain	The largest encyclopedia in the Kazakh language, an indicator of language development in the open-source environment
National Corpus of the Kazakh Language (https://qazcorpus.kz/index.php)	Academic domain	A representative corpus reflecting the normative state of the language

In our view this selection ensures coverage of both government (top-down) and user (bottom-up) aspects of language digitalization, and allows us to assess not merely the presence of the language, but its functional load in various niches. However, an assessment of the functional use of the Kazakh language on these platforms was not the objective of this study.

Data collection was carried out primarily between October and December 2025. Interface analysis was conducted based on the following parameters:

1. presence/absence of a language switch;
2. default language;
3. completeness of the interface translation;
4. proportion of content in the Kazakh language (% of total volume);
5. functional equivalence (whether the functions match in both language versions);
6. visual and semiotic representation: use of Kazakh script (Cyrillic/Latin); presence of visual markers of national identity.

In order to construct an overall picture of the public discourse in which narratives concerning language digitalisation are formed, the research methodology has been supplemented by an analysis of additional media materials. In particular, the subject of the study was the transcription of an interview with the Minister of Science and Higher Education of the Republic of Kazakhstan S. Nurbek as an example of expert and political discourse. This interview is considered not as a statistically representative sample, but as an expert interview (target sample), since the respondent is a government top manager and his statements can be qualified as the conclusions of a specialist whose job responsibilities include the implementation of the issues under study. This allows us to uncover hidden institutional mechanisms that are invisible when analyzing documents. The text was obtained by downloading from an open source (Bilimdi el, 2026). Coding was carried out by the method of inductive thematic analysis in the MaxQDA program.

## Results

### State programmes as the conceptual foundation of Kazakhstan’s language policy

A critical discourse analysis of policy documents revealed key changes in the goals and content of Kazakhstan’s official language policy over the period from 2010 to the present, as well as trends driven by the digital transformation of society. A general frequency and contextual analysis of the vocabulary in the texts of the State Programme for the Development and Functioning of Languages in the Republic of Kazakhstan for 2011–2019 (2011), the State Programme for the Implementation of Language Policy in the Republic of Kazakhstan for 2020–2025 (2019), and the Concept for the Development of Language Policy in the Republic of Kazakhstan for 2023–2029 (2023), enabled the identification of key words and phrases reflecting the content, ideology and terminological core of the policy documents (Table 2).

**TABLE 2:** Key vocabulary of the texts of state program documents on the language policy of Kazakhstan (for 2011–2019, 2020–2025, 2023–2029).

The State Programme for the Development and Functioning of Languages in the Republic of Kazakhstan for 2011–2019	State Programme for the Implementation of Language Policy in the Republic of Kazakhstan for 2020–2025	Concept for the Development of Language Policy in the Republic of Kazakhstan for 2023–2029
Keywords and phrases		
State language policy, the Kazakh language as the state language, the trinity of languages, multilingualism, language planning, social cohesion, the linguistic environment, inter-ethnic harmony, national unity, civic identity	Kazakh as the official language, official language policy, identity, multilingualism, language use, language behavior, linguistic identity, linguistic responsibility, digitalisation of the language sphere, implementation of language policy, expansion of the public functions of the Kazakh language, competitiveness of the official language	Official language, language standards, compliance with standards, monitoring of compliance, linguistic norms and rules; visual space, information placement, visual information, signs and signposts, linguistic environment; teaching of the official language, teaching materials, language competence, educational resources; language of science, terminology work, creation of a terminology database; digital environment, internet resources, electronic platforms, online content, digital resources

In terms of topic and terminology, the phrases in the first document can be classified as state-regulatory and civil language, reflecting language policy aimed at harmonizing linguistic interaction. Digital issues are only briefly addressed in the program text, through individual words characterizing areas of language use: *websites*, *translations*, and *ICT in education*.

The themes and terminology of the second document have undergone changes: While key concepts such as *the state language, state language policy, identity*, and *multilingualism* have been retained, new phrases have emerged, including *language practice, language behavior, linguistic identity, linguistic responsibility*, and *digitalisation of the linguistic sphere*. It is evident that state-normative discourse is being replaced by social and applied discourse (*implementation of language policy, expansion of the public functions of the Kazakh language, competitiveness of the state language*). It is notable that the State Programme, for the first time, refers to the digitalisation of the language sphere, using terms such as “*media space*,” “online communication,” and “*language of modernisation*.”

The Concept for the Development of Language Policy for 2023–2029 (2023) identifies the following thematic clusters: regulatory and legal (*fundamental principles of language policy development; implementation of language policy based on the linguistic environment)*, educational (*improvement of language teaching methods*), socio-cultural (*language of visual information; enhancing the linguistic culture of society*), scientific *(promoting the use of the Kazakh language as the language of science*), and digital (*increasing the proportion of content in the Kazakh language*). The presence of particular combinations of words become more evident: “visual space,” “placement of information,” “visual information,” “signs and signposts,” “linguistic environment”; “teaching the state language,” “educational resources”; “the language of science,” “terminological work,” “creation of a terminological database”; “the digital environment,” “internet resources,” “electronic platforms,” “online content,” “digital resources.”

A comparative discourse analysis of government programmes in the field of Kazakhstan’s language policy reveals a shift from a normative and declarative approach to a policy of legal regulation focused on the digital realm.

In order to identify the digital component of Kazakhstan’s language policy, a thematically focused frequency analysis of texts was conducted in addition to a general frequency analysis. For the purpose of this study, 25 marker words related to the thematic cluster of “Digitalization” and with a frequency of ≥4 units were selected (see Table 3).

**TABLE 3:** Comparative analysis of the frequency of use of keywords from the “Digitalisation” lexical-semanic group in the texts of government programmes.

No	Keywords	State Programme for the Development and Functioning of Languages in the Republic of Kazakhstan for 2011–2019	State Programme for the Implementation of Language Policy in the Republic of Kazakhstan for 2020–2025	Concept for the Development of Language Policy in the Republic of Kazakhstan for 2023–2029
Number of occurrences of words in the document				
1	Development	33	52	97
2	Information/informational	25 (15/10)	113 (67/46)	111 (79/32)
3	Implementation	8	13	19
4	Technology/IT	7 (7/0)	21 (14/7)	48 (32/16)
5	Content	7	19	30
6	Electronic	5	13	26
7	Communication/communicative	2 (2/0)	22 (19/3)	8 (7/1)
8	Resource	3	14	11
9	The internet	4	17	35
10	Innovation/innovative	2 (0/2)	10 (5/5)	8 (4/4)
11	Remote	3	1	3
12	Data	2	3	4
13	Online	2	13	12
14	Portal	2	5	12
15	Search/search-related	1 (1/0)	5 (1/4)	6 (4/2)
16	Website	1	11	22
17	Network	1	2	15
18	Service	1	7	4
19	Digital/digitalization	1 (1/0)	4 (4/0)	27 (23/4)
20	Corpus	0	32	40
21	Modernization	0	14	7
22	Computerization	0	5	0
23	Site	0	3	1
24	Mobile	0	2	13
25	Interface	0	2	4

The keywords listed in Table 3 form a single semantic-thematic cluster associated with digitalisation, technological advancement and the emergence of a new information environment. Consequently, these keywords can be readily categorized into thematic subgroups. For instance, the terms “innovation,” “technology,” “modernisation,” “implementation,” “informatisation” and “digital/digitalisation” can be grouped into the thematic block “Technological Progress,” whilst the terms “information,” “data,” “communication,” “network,” “website” and “portal” can be grouped into the block “Information and communication (including online communication).”

The table demonstrates a consistent upward trend in the frequency of digital and technology-related vocabulary, which indicates an undeniable expansion of the thematic scope of Kazakhstan’s language policy. This suggests that the discourse of state language planning is shifting toward digital transformation (for instance, whilst the word “digital” appears only once in the earliest document analyzed, it appears four times in the second and 27 times in the current one; the words “technology” and “IT” appear 7, 21, and 48 times, respectively). It is noteworthy that the term “transformation” is present in the final two documents: The frequency of occurrence of the words in question was determined to be one and two times, respectively. However, they did not make it into the list of 25 marker words, as words with a frequency of use <4 were not taken into account. Despite the content analysis of the programmes’ texts revealing no use of the terms “digital language policy” or “digital language regulation” – terms that are gradually entering academic and official terminology – language policy is presented as part of a new digital paradigm in the last two documents. This is reflected in the increased use of words denoting new objects of regulation (website, portal, network, service, corpus, etc.), technological tools and infrastructure (technology/IT, resource, internet, mobile, interface, etc.). The increase in the number of words denoting processes and describing the outcomes of activities (development, implementation, modernisation, innovation, informatisation, digitalisation, etc.) indicates a shift in state policy from declarations to action. The rise in the frequency of words marking digitalisation is no coincidence: it reinforces the new priorities of Kazakhstan’s state language policy.

The digital and technology-oriented focus of language policy in recent years has enabled the identification and analysis of the contexts in which key terms are used. For instance, the term “implementation” is used in the following phrases in the 2011–2019 Programme: *introduction of a system for assessing proficiency in the state language, introduction of distance learning, introduction of digital television and radio broadcasting, introduction of new information technologies into the process of learning the state language*. In the 2020–2025 document, the term is used in the following phrases: *introduction of actively used sector-specific loan terms into the national terminology system, introduction of the Kazakh language into the “Google Translate” and “Yandex Translator” translation services*, and in the Concept for the Development of Language Policy in the Republic of Kazakhstan for 2023–2029 (2023), the term is used as follows: *integration of the Kazakh language into the “Steam” digital store, integration of the Kazakh language into the international information space, integration of the Kazakh language into the virtual space, and efforts to establish the state language as one of the competitive, sought-after languages in the online space*. The word “resource” appears only three times in the first document, of which just once in the phrase “internet resource”; in the second document, it appears 14 times, of which eight are in combination with the word “internet”; in the third, it appears 11 times, and in all cases, it refers to “internet resources.”

Consequently, the conducted frequency content analysis of the text of program documents in the field of language policy (2011–2019, 2020–2025, 2023–2029) allows for the identification of a trend of gradual transition from a normative-regulatory model focused on the establishment of quotas, standards, and requirements for the use of the state language, to an infrastructural model involving the creation of digital resources, platforms, and technological foundations for the full functioning of the Kazakh language in the digital environment. This shift is being operationalised through the development of a system of specialized digital platforms, each of which ensures the presence of the Kazakh language in a specific functional sphere. In the following sections, an examination will be conducted of the key platforms that reflect the various levels of implementation of the language infrastructure policy. These platforms will be considered in turn, beginning with basic linguistic resources that provide the technological foundation, and concluding with mass digital content platforms and the infrastructure of public administration. In between these two extremes, the focus will be on educational platforms that integrate the language into the sphere of academic knowledge.

### Digital platforms as tools for implementing Kazakhstan’s language policy

The National Corpus of the Kazakh Language (NCKL; QazCorpus, 2026) constitutes the core element of the language policy infrastructure model, providing the technological foundation for natural language processing and the development of domestic NLP technologies. The legal status and functions of this corpus are defined by Order No. 227 of the Minister of Science and Higher Education of the Republic of Kazakhstan dated 30 April 2025. It is asserted in this order that the NCKL serves as the basis for scientific, educational, and practical research in the field of Kazakh language and linguistics, as well as the development of language technologies, language models, machine translation systems, and AI. Strategic documents of Kazakhstan’s government agencies on digital development have highlighted the lack of realizing the full potential of the Kazakh language. The development of the National Corpus of the Kazakh Language (QazCorpus, 2026) was initiated to address these issues. Implemented by specialists from the A. Baitursynov Institute of Linguistics, the project is a fundamental initiative to create a digital annotated computer system that includes extensive information on the Kazakh language and serves as an IT resource for scientific research and education. The corpus performs an important function in supporting and preserving the language, ensuring its viability in the digital environment through the automation of lexicographic processes and the creation of a reference database of modern word usage.

The corpus interface facilitates access to 250-million-word tokens, which are structured into 22 specialized subcorpora. The platform’s key feature is its deep linguistic annotation, in which all words are annotated with phonetic (orthoepy, syllabic structure), phonological (phonemic characteristics), lexical (dictionary meanings), and morphological (lemmatization, parts of speech) annotations. The corpus structure encompasses all functional styles (colloquial, journalistic, scientific, formal-business, and literary); the oral subcorpus contains audio and video recordings with transcriptions; the historical subcorpus contains written records of various graphic systems; and the dialectological subcorpus captures regional linguistic features. Of particular importance are parallel subcorpora (including a six-character parallel corpus with texts in Kazakh, English, Turkish, Uzbek, Uyghur, and Azerbaijani languages), as well as specialized resources – terminological, onomastic, a corpus of proverbs and sayings, and a corpus of errors. The NKKL’s contribution to the implementation of language policy is evident in its establishment of a digital infrastructure for the development of language technologies. The availability of structured, labeled corpus data in the Kazakh language is a prerequisite for technological independence in AI, enabling the creation of domestic NLP solutions independent of foreign language models trained primarily on English-language data.

In addition to technological infrastructure, educational platforms that integrate the Kazakh language into the global educational ecosystem should be considered. The initiative to adapt the global Coursera platform’s courses into Kazakh language versions, launched in 2023 by order of the Ministry of Science and Higher Education of the Republic of Kazakhstan, represents a mechanism for integrating the state language into the existing international educational platform (Coursera, n.d.-b).

According to official data (Government of the Republic of Kazakhstan, 2026), over 12,000 courses translated into Kazakh are available to users on the platform, of which students have completed approximately 4,700 different educational programs (40% of the total number available within the project). During the 3-year implementation phase of the project, users have earned 346,443 certificates. The dynamics of average monthly indicators demonstrate a steady increase: from 6,083 in 2023 to 32,295 in 2025.

The structure of the most popular courses within the project shows the diversity of disciplinary areas: from courses on communications and time management [“21st century workplace relationships” – 5,392 certificates; “Time management for professional productivity” – 2,155 certificates, “Word Forms and Simple Present Tense” – 2,786 certificates, “Programming for Everybody (Getting Started with Python)” – 2,706 certificates, “Learning How to Learn: Powerful mental tools to help you master tough subjects” – 2,669 certificates, “Artificial Intelligence (AI) for Everyone” – 2,118 certificates, “Introduction to Microsoft Excel” – 2,104 certificates, “Entrepreneurship I: Building the Foundations of a Business” – 2093 certificates]. It is noteworthy that among the popular courses within the project there are both fully adapted into the Kazakh language and English-language courses with Kazakh-language support.

The dynamics of the academic community’s involvement in the project demonstrate rapid institutional uptake: whilst 25 universities participated in the programme in 2023, in 2025 the number reached 95. Over a period of 3 years, the ecosystem has reached more than 167,000 students, who have confirmed their competencies by obtaining over 288,000 certificates. It is noteworthy that within the specialized AI Sana stream, focused on artificial intelligence, approximately 47,000 certificates were issued. These figures are consistent with data from the Global Skills Report 2025, in which Kazakhstan secured 37th place in the global skills ranking (Coursera, n.d.-a). However, it should be noted that these statistics are indicative of an institutional challenge: the competitiveness of the workforce is increasing at a faster rate than the technological maturity of the national AI ecosystem, in which the country ranks only 51st (Global Skills Report, 2025). The integration of the Kazakh language into the Coursera ecosystem provides access to international academic content in the state language. The adaptation of courses and the development of an appropriate terminology database contribute to the use of the Kazakh language in the fields of higher education, science and technology.

In addition to the international educational platform, the national project “New Humanitarian Knowledge. 100 New Textbooks in the Kazakh Language” (100 oqulyq, 2026) should be considered. This project is an illustration of the implementation of the language policy through the successive translation of 100 world textbooks in humanitarian disciplines (economics, management, philosophy, sociology, psychology, religious studies, etc.) into the Kazakh language, with the objective being to ensure access to advanced academic knowledge in the state language (National Bureau of Translations, n.d.). As part of the project, 100 textbooks were translated into Kazakh, published in print runs of 10,000 copies each, and distributed to all universities across the country. Digital versions of the textbooks, in conjunction with a series of video lectures based on them, are available on the Open University of Kazakhstan platform (OpenU, 2026). The platform offers complimentary access to the translated textbooks and online courses developed by leading Kazakhstani universities. The platform’s library section contains translations into Kazakh of the most distinguished books from universities around the world.

The “100 New Textbooks” project and the Open University platform contribute to the development of a national educational infrastructure that provides access to the world’s academic heritage in the Kazakh language. This resource serves as a foundation for the development of humanities education in the country and the dissemination of cutting-edge academic knowledge. In contradistinction to the flexible and market-oriented partnership with Coursera, the “New Humanities Education: The 100 New Textbooks” in Kazakh initiative (100 oqulyq, 2026) can be regarded as an example of state planning that is subject to strict regulation, with the aim of addressing a shortage of educational content. This initiative signifies more than a mere publishing project; it is a deliberate effort to transfer academic standards and research paradigms from globally renowned universities to the context of Kazakhstani practice. Through the translation of foundational works, the normative basis has been laid for the Kazakh language to function as a fully-fledged tool of higher academic scholarship.

Another indicator of the Kazakh language’s presence in global digital practices is the Kazakh-language version of Wikipedia (Qazaqsha Wikipedia) – a global online resource that has been developed by a community of users and volunteers in the Kazakh language since 2022 (Wikimedia Foundation, 2026).

The main page of the section displays the complete localisation of the interface: the navigation menu, editing tools, categories and service pages are presented in Kazakh. The statistics block, located in the right-hand portion of the screen, meticulously records the quantitative indicators of the development of the section. At the time of access, the section comprised 241,858 articles covering a wide range of subjects, with approximately 2 million edits and a membership of over 10,000 registered participants, of which 100–150 are actively engaged in editing (Wikidata, 2026). This structural element suggests that the platform functions as an instrument of accumulation, systematization and updating of knowledge in the state language.

In contrast to institutional and educational platforms that function within the framework of state directives, Kazakh Wikipedia ensures the presence of the Kazakh language in the domain of encyclopedic knowledge and mass digital content by facilitating the creation of digital content by the language community. The platform functions as a repository of reference and encyclopedic knowledge in the Kazakh language, thereby establishing a specialized information resource within the network. The technological structure of the Kazakh version of Wikipedia is of fundamental importance for language policy. The content of the resource supports automatic text conversion in three graphic systems: Cyrillic, Latin and Arabic, which allows it to take a stable position among other language versions in the global network. The mechanism of automatic conversion simultaneously performs educational, cultural and integration functions. It facilitates the adaptation of users to Latin, preserves the connection with the historical written heritage and provides access to the resource for various groups of speakers of the language, including the diaspora outside of Kazakhstan.

The effectiveness of this project is determined not by the number of texts translated, but by the extent to which they are incorporated into educational and digital practices. The pivotal indicators in this context pertain to the utilization of textbooks in university courses, the frequency with which they are cited, and their integration into digital educational and language technologies. It is evident that the transition from one-off initiatives to a sustainable model can only be achieved through the institutionalization of terminological resources, the formal recognition of the status of translated textbooks, and the extension of the project to the natural and technical sciences. In this case, the translation of academic knowledge could become a key driver of the development of the Kazakh language within the higher education system and the digital environment. Nevertheless, the transformative impact of the project remains ambiguous and requires critical evaluation. The establishment of a systematic academic corpus that establishes benchmarks for terminological and stylistic norms of the Kazakh language in the field of online education is an indisputable achievement. However, the lack of transparency in the translation methodology and terminology harmonization procedures limits the integrity of the corpus being developed and creates a risk of fragmentation and formal calquing of terms (see Kaplan and Baldauf, 1997; Baldauf, 1989).

The eGov.kz portal (eGov.kz, 2026) is of particular significance in the context of the strengthening of the functioning of the Kazakh language in the digital space.

This portal represents a digital infrastructure through which citizens interact with the state. The portal interface includes a switch for language versions (KAZ, RUS, ENG) at the top of the page. In the central part of the screen there is a classification of public services by thematic blocks and life situations: “Family,” “Health Care,” “Education,” “Business,” “Employment and employment” and others. Such a structure shows that the language policy is implemented not just through the translation of the interface, but through the adaptation of bureaucratic and administrative terminology, turning the Kazakh language into a full-fledged instrument of interaction between citizens and state institutions in the digital environment.

As the analysis of the portal demonstrated, language support is implemented not at the level of formal duplication of the interface, but as a systemically integrated mechanism of administrative and legal interaction. The platform’s interface is available in three languages (Kazakh, Russian and English). The Kazakh version is presented first, reflecting the official prioritization of the state language. The navigation structure, electronic forms, service notifications and official content have all been fully adapted to Kazakh. It should be noted that news materials on the main page are published in the state language and cover not only the strategic directions of the development of digital services, but also reports on the functioning of individual state platforms. It is evident that the promotion of the Kazakh language as a working tool for the implementation of legally significant actions, including obtaining certificates, submitting applications and drafting documents, will be a key benefit.

In contrast to educational and encyclopedic platforms, where translation is primarily focused on facilitating access to knowledge, in the case of state digital services, it is related to the provision of legally and administratively significant actions. The user is required to utilize Kazakh terminology when completing official forms and interpreting instructions, thereby facilitating the integration of the language into their daily digital practices. Another key indicator of the institutional status of the language is the presentation of user statistics. Activity indicators, including the total number of visitors per month, day and the current number of users on the portal, are displayed in both Kazakh and Russian languages. The data do not indicate the user’s language preferences, but they confirm the status of the state language as the language of official digital reporting and public communication. The institutional aspect of the language policy is implemented on the eGov.kz portal by integrating the state language into the digital infrastructure of the state administration. This ensures the full functioning of the Kazakh language in the field of providing public services and everyday interaction between citizens and the state.

The digital platforms examined demonstrate diverse yet complementary models for implementing Kazakhstan’s digital language policy. The Kazakh version of Wikipedia fosters a volunteer-driven corpus-based environment and ensures the language’s presence in the global information space. Coursera Qazaqstan implements an educational model with limited institutional sustainability, where the Kazakh language is incorporated primarily through translation and subtitles. The eGov.kz (2026) platform represents a legally binding model, within which the Kazakh language functions as an essential tool for legally significant digital interaction. In one way or another, they all serve as instruments of digital linguistic sovereignty.

The digital platforms examined demonstrate diverse yet complementary models for implementing Kazakhstan’s digital language policy. The Kazakh version of Wikipedia fosters a volunteer-driven corpus-based environment and ensures the language’s presence in the global information space. Coursera Qazaqstan implements an educational model with limited institutional sustainability, where the Kazakh language is incorporated primarily through translation and subtitles. The eGov.kz (2026) platform represents a legally binding model, within which the Kazakh language functions as an essential tool for legally significant digital interaction. In one way or another, they all serve as instruments of digital linguistic sovereignty.

## Discussion

An analysis of Kazakhstan’s strategies for the formation of the national infrastructure of knowledge reveals a model in which the language policy is implemented through institutional control over digital instruments. The results of critical discourse analysis of official documents (2010–2025) demonstrate that language is constructed not only as an identity marker, but also as an infrastructural resource. This approach aligns with the principles of digital inclusion as outlined by UNESCO (2021). However, it is important to note the Kazakh specificity, which manifests in the prioritization of state platforms over market mechanisms. In contrast to contexts in which local languages are preserved in a symbolic capacity, projects such as the National Corpus of the Kazakh language or Coursera in Kazakh are aimed at functional use of the language in management, education and professional communication. The choice of Coursera in the Kazakh language as a case study is predicated on the fact that this represents one of the few instances in which the state initiative of digitalisation of higher education and language policy intersect directly and in a documented manner: the translation of university courses into Kazakh is recorded in programme documents as a discrete task. This cooperation enables the realization of two functions of language planning: firstly, the implementation of prestige planning, due to the association of the Kazakh language with an authoritative international educational platform; and secondly, the execution of corpus planning, since the translation of courses necessitates the development of new academic terminology in areas where Kazakh-language materials were practically non-existent prior to this. This trajectory is analogous to the concept of “Language Policy 4.0” (Kelly-Holmes, 2023), yet it entails a shift in emphasis from automation to the sovereignty of digital media.

Institutional analysis of interfaces (e.g., eGov.kz, Wikipedia, educational platforms) confirms Spolsky’s (2004) thesis on status planning through digital presence. The strengthening of the position of the Kazakh language is achieved through its anchoring in recognized services. The prestige of the language is maintained not so much through media consumption as through institutional integration, a factor which distinguishes Kazakhstan from the models described by Eke and Salawu (2026). It is evident that a substantial volume of content, along with the presence of indicators of visits to the Kazakh version of Wikipedia, signifies the active presence of the Kazakh language in the digital sphere. For instance, since 2011, the number of articles has increased by over 100,000, surpassing 200,000. This growth can be attributed to the collaborative efforts of volunteers, editors, communities, and the participation of universities, as well as partial support for translation initiatives utilizing materials from national encyclopedias (Wikidata, 2026). However, the project has a number of significant structural limitations. Competition with Russian-language and English-language Wikipedia has led to users turning to larger and more elaborate sections when searching for specialized information, which has resulted in an influx of readers and new editors. A significant structural limitation is the non-formation of a stable base of authoritative sources in the Kazakh language, necessary for the preparation of encyclopedic materials, primarily in scientific and technical fields. The situation is complicated by the lack of unified and generally accepted terminological standards in the field of high technologies and modern sciences, which complicates the formation of meaningfully verified encyclopedic content. In the long-term perspective, the value of this project is determined not only by the volume of content, but also by the degree of its integration into educational, scientific and technological practices.

Nevertheless, despite the state’s successful implementation of large-scale technological measures at present, it has become evident that the prevailing technocratic narrative effectively overshadows more intricate sociolinguistic concerns. Despite official statements about strengthening the Kazakh language as a unifying factor for all ethnic groups living in Kazakhstan, practice shows that users, especially the most active part of them – the youth – choose the language based on its socio-pragmatic possibilities, and in the digital environment - also taking into account its resource (educational, professional, informational) potential (Zharkynbekova et al., 2025a,b; Galiyeva et al., 2024). As Yessenbekova et al. (2025) observes, despite the high level of support for digitalisation as a language preservation tool among young people (93.6%), more than four users (26.2%) decline Kazakh-language resources due to the perceived inconvenience of interfaces.

Digital inequality poses a threat to linguistic diversity, but the strategic use of digital technologies can help prevent digital extinction. This aligns with the findings of research on the efforts of states and scientific communities to preserve languages using digital resources, natural language processing (NLP), or language learning materials created by large language models (LLMs) (Giglitto et al., 2023). It is evident that digital technologies facilitate the documentation of language and cultural heritage (Chernyavskaya and Zharkynbekova, 2024). These questions are of particular relevance for Kazakhstan, given the historically developed language situation (Tlepbergen et al., 2023, 2025; Zharkynbekova et al., 2025a; Fazylzhanova and Piyazbaeva, 2024). In the contemporary context of the Kazakh language’s existence, when its position is significantly influenced by other languages (most notably Russian) (Suleimenova, 2020; Chernyavskaya and Zharkynbekova, 2019), the real role of all implemented projects related to the support of the state language is associated not with the fact of their existence, but the quality of their concept, content, and functional orientation. It is imperative that their content is connected with real communicative practice. For instance, despite the presence of institutional support, an unspoken division persists: the Kazakh language is associated with official services (e.g., eGov, state portals), while Russian dominates in commercial, entertainment and informal digital spaces. It is important to note that by ignoring these spontaneous forms of communication in favor of strict normativity, a barrier is created between the official digital environment and live language practice. Without an examination of the motivations behind the utilization of language upon transitioning between different domains, investments in infrastructure may inadvertently solidify the Kazakh language’s position as an interface language rather than a language that is widely spoken in day-to-day life. Furthermore, individuals possess divergent linguistic repertoires and digital habits, which are contingent on their age. For young people, for example, the Kazakh language is a marker of identity, but this does not mean that it is also a tool for technical communication (Galiyeva et al., 2024; Zharkynbekova et al., 2025a). It is imperative to examine which digital practices contribute to the transformation of symbolic support into functional use.

As indicated by the aforementioned points, infrastructure sovereignty may continue as a mere formality unless state-run platforms are used for organic communication. This emphasizes the importance of interdisciplinary collaboration: mitigation of algorithmic and interface bias necessitates the involvement of linguists during the development phase rather than post-implementation. In addressing the question posed in this study, it is crucial to emphasize that the sustainability of digital sovereignty is contingent not only on the quantity of resources created, but also on their capacity to adapt to the actual, often hybrid, forms of users’ linguistic behavior.

From a comparative perspective, the case of Kazakhstan demonstrates that digital sovereignty is not a universal model. The viability of this model is contingent upon the efficacy of its technological implementation and the capacity to adapt global formats to the unique characteristics of an agglutinative language. The strategy of developing a national knowledge infrastructure helps to ensure that the language remains within its functional sphere, but raises the question of scalability: successful solutions must be translated into regional practices relevant to other multilingual societies.

## Limitations

Notwithstanding the contribution made, it is necessary to consider the methodological limitations of this study. The digital platforms delineated in the “Results” section establish the institutional and technological conditions for the Kazakh language to function in the digital environment. Nevertheless, the extent of their actual impact on language practices, users’ choice of language and the formation of linguistic identity remains a subject for further research. The absence of data concerning user behavior – including their preferences regarding the choice of language version, the frequency of use of Kazakh-language content, and its perception by different age and social groups – prevents definitive conclusions from being drawn about the actual impact of digital infrastructures on language preservation. This issue suggests directions for future empirical research, in which the institutional analysis presented in this paper could be supplemented by sociological and psycholinguistic methods.

## Conclusion

The study confirms that Kazakhstan’s language policy is undergoing a transition from a normative-status-based approach to an infrastructural one. The pivotal mechanism for language preservation is evolving to encompass not only the declaration of status but also state control over digital data processing platforms. The model of “digital linguistic sovereignty” that was identified during the analysis suggests that the viability of a language on the global network is contingent upon the availability of national tools for managing content and interfaces. This finding contradicts the hypothesis that digitalisation leads to the dominance of global languages. Institutional support can facilitate the sustenance of local languages as functional entities within governance and education.

The theoretical contribution of this work lies in expanding the concept of language planning. The linguistic security is now determined not only by the availability of a language, but also by the quality of the user experience. The observed discrepancy between the political goals and interface usability indicates the necessity of a review of existing development standards: localisation should address the logic of interaction, rather than merely the lexical layer. Unless the functional limitations of digital resources are addressed, government policy risks remaining merely declaratory, despite high demand, for example, from young people.

In future research, a comparative analysis of digital sovereignty models in the post-Soviet space, particularly in Central Asian countries, will facilitate the identification of common patterns for the formation of language resilience in the face of technological inequality.

## Data Availability

The original contributions presented in this study are included in this article/supplementary material, further inquiries can be directed to the corresponding author.

## Footnotes

1
http://adilet.zan.kz/

2
http://prg.kz/

## References

[B1] Alkebaeva D. Sultan E. (2025). Qazaq tłldł zhasandy intellekttegł lingvistikalyκθzgerłster. [Linguistic changes in Kazakh-language artificial intelligence]. *Eur. J. Philol. Sci. Educ.* 198 19–33. 10.26577/EJPh202519822

[B2] Androutsopoulos J. (2013). Networked multilingualism: Some language practices in Facebook and their implications. *Intern. J. Biling.* 19 185–205. 10.1177/1367006913489198

[B3] Androutsopoulos J. (2014). “Mediatization and Sociolinguistic Change,” in *Mediatization and Sociolinguistic Change*, ed. Androutsopoulos J. (Berlin: De Gruyter), 3–28. 10.1515/9783110346831

[B4] Baker P. (2006). *Using Corpora in Discourse Analysis.* Boston, MA: Continuum.

[B5] Baldauf R. B. (1989). Language planning: Corpus planning. *Ann. Rev. Appl. Ling.* 10 3–12. 10.1017/S0267190500001173

[B6] Bender E. M. Gebru T. McMillan-Major A. Shmitchell S. (2021). “On the Dangers of Stochastic Parrots: Can Language Models be Too Big?,” in *Proceedings of the 2021 ACM Conference on Fairness, Accountability, and Transparency*, (New York, NY: ACM).

[B7] Bilimdi el (2026). *Sayasat Nurbek & Lyazzat Turkestanova: A Conversation About the Future of Higher Education and AI.* San Bruno, CA: Youtube.com.

[B8] Bourdieu P. (1991). Language and Symbolic Power. Cambridge: Polity Press.

[B9] Bowe B. J. Freedman E. Blom R. (2012). Social media, cyber-dissent, and constraints on online political communication in Central Asia. *Central Asia Caucasus* 13 144–152.

[B10] Chernyavskaya E. Zharkynbekova S. K. (2024). Code switching patterns in Kazakh-Russian hybrid language practice: An empirical study. *Train. Lang. Culture* 8 9–19. 10.22363/2521-442X-2024-8-2-9-19

[B11] Chernyavskaya V. E. Zharkynbekova S. K. (2019). Linguistic and social construction of national university identity: Kazakh and Russian Universities’ mission statements. *Bull. St Petersburg University. Lang. Liter.* 16 304–319. 10.21638/spbu09.2019.210

[B12] Concept for the Development of Language Policy in the Republic of Kazakhstan for 2023–2029 (2023). *Approved by the Decree of the Government of the Republic of Kazakhstan.* Available online at: https://youthlib.mirea.ru/ru/resource/5020 (accessed October 16, 2023).

[B13] Cooper R. L. (1989). *Language Planning and Social Change.* Cambridge: Cambridge University Press.

[B14] Couldry N. Mejias U. (2022). Data colonialism: Rethinking big data’s relation to the contemporary subject. *Quest. Commun.* 42 205–221. 10.4000/questionsdecommunication.29845

[B15] Coursera (n.d.-a). *Kazakhstan Modernizes Educatoion and Transforms Economies with Coursera.* Available online at: https://www.coursera.org/enterprise/resources/casestudy/mshe

[B16] Coursera (n.d.-b). *Kazakhstan’s Ministry of Science and Higher Education Drives Digital Learning at Scale with Coursera.* Available online at: https://www.coursera.org/enterprise/resources/casestudy/mshe-award-winner

[B17] Cunliffe D. (2007). “Minority Languages and the Internet: New Threats, New Opportunities,” in *Minority Language Media: Concepts, Critiques and Case Studies*, eds Cormack M. Hourigan N. (Bristol: Multilingual Matters), 133–150.

[B18] Dave B. (2007). *Kazakhstan: Ethnicity, Language and Power.* Milton Park: Routledge, 10.4324/9780203014899

[B19] eGov.kz. (2026). *Electronic Government Portal of the Republic of Kazakhstan.* Available online at: https://egov.kz/cms/kk (accessed January 25, 2026).

[B20] Eisenberg L. (2021). *Janasozdik: Codifying an Inclusive Kazakh Slang.* Cambridge, MA: Harvard University.

[B21] Eke I. W. Salawu A. (2026). Impact of digital media on language revitalization: The case of BBC News Igbo. *Front. Commun.* 11:1707701. 10.3389/fcomm.2026.1707701

[B22] European Parliament (2018). *European Parliament resolution of 11 September 2018 on Language Equality in the Digital Age (2018/2028(INI)).* Strasbourg: European Parliament.

[B23] Fairclough N. (1992). *Discourse and Social Change.* Cambridge: Polity Press.

[B24] Fazylzhanova A. Piyazbaeva A. (2024). Jazykovaja situacija v Kazahstane: sravnitel’nyj analiz sovetskogo i nezavisimogo periodov [Language situation in Kazakhstan: A comparative analysis of the Soviet and independent periods]. *Tiltanym* 4 116–134. 10.55491/2411-6076-2024-4-116-134

[B25] Fedorova K. Tšuikina N. (2024). Multilingual tallinn: People and languages in the urban space. *J. Eur. Stud.* 15 192–212. 10.1177/18793665241269048

[B26] Fierman W. (2006). Language and education in post-soviet kazakhstan: Kazakh-medium instruction in urban schools. *Russ. Rev.* 65 98–116. 10.1111/j.1467-9434.2005.00388.x

[B27] Fishman J. A. (1991). *Reversing Language Shift: Theoretical and Empirical Foundations of Assistance to Threatened Languages.* Bristol: Multilingual Matters.

[B28] Floridi L. (2020). The fight for digital sovereignty: What it is, and why it matters, ethical framework for the information society. *Philos. Technol.* 33 369–378. 10.1007/s13347-020-00423-6 35194548 PMC8848320

[B29] Galiyeva B. K. Zharkynbekova S. K. Ayupova G. K. (2024). Linguistic manifestations of self-identification of Kazakhstani student youth (on the basis of essays). *Terra Ling.* 15 18–35. 10.18721/JHSS.15202

[B30] Giglitto D. Ciolfi L. Lockley E. Kaldeli E. (eds) (2023). *Digital Approaches to Inclusion and Participation in Cultural Heritage: Insights from Research and Practice in Europe*, 1st Edn. Milton Park: Routledge.

[B31] Global Skills Report (2025). *The Report at a Glance.* Mountain View, CA: Global Skills Report.

[B32] Goloburda M. Laiyk N. Turmakhan D. Wang Y. Togmanov M. Mansurov J. et al. (2025). Qorǵau: Evaluating LLM safety in kazakh-russian bilingual contexts. *arXiv* [Preprint] 10.48550/arXiv.2502.13640

[B33] Government of the Republic of Kazakhstan (2026). *Distancionnoe Obuchenie [Distance learning].* Available online at: https://egov.kz/cms/ru/articles/distance_learning (accessed January 10, 2026).

[B34] Grenoble L. A. Whaley L. J. (2006). *Saving Languages: An Introduction to Language Revitalization.* Cambridge: Cambridge University Press.

[B35] Kaplan R. B. Baldauf R. B. (1997). *Language Planning: From Practice to Theory.* Bristol: Multilingual Matters, 10.21832/9781800418059

[B36] Keet C. M. Khumalo L. (2023). Contextualising levels of language resourcedness that affect NLP tasks. *arXiv* [Preprint] 10.48550/arXiv.2309.17035

[B37] Kelly-Holmes H. (2023). Language policy 4.0: Agency in an increasingly automated future. *Chinese J. Lang. Policy Plan.* 8, 14–25. 10.19689/j.cnki.cn10-1361/h.20230101

[B38] Kirchmeier S. (2020). Trends in European language policies with a view to language technology. *Bendrinë Kalba* 93 1–23. 10.35321/bkalba.2020.93.07

[B39] Kornai A. (2013). Digital language death. *PLoS One* 8:e77056. 10.1371/journal.pone.0077056 24167559 PMC3805564

[B40] Kucherbayeva D. Smagulova J. (2026). From policy to practice: Pragmatic acceptance of translanguaging among secondary school teachers in Kazakhstan. *Pedagogy Teach. Methods* 2 197–210. 10.47344/twt5n268

[B41] Kuulmets H.-A. Purason T. Luhtaru A. Fishel M. (2024). *Teaching Llama a New Language Through Cross-Lingual Knowledge Transfer. Findings of the Association for Computational Linguistics: NAACL.* Mexico: Association for Computational Linguistics.

[B42] Kwet M. (2019). Digital colonialism: US empire and the new imperialism in the Global South. *Race Class* 60 3–26. 10.1177/0306396818823172

[B43] Liddicoat A. J. Kirkpatrick A. (2020). Dimensions of language education policy in Asia. *J. Asian Pacific Commun.* 30 7–21. 10.1075/japc.00043.kir

[B44] Migge B. Schneider B. (2025). The material making of language as practice of global domination and control: Continuations from European colonialism to AI. *AI Soc.* 40 6059–6071. 10.1007/s00146-025-02389-5

[B45] Ministry of Education and Research of the Republic of Estonia (2018). *The Language Technology Research and Development Program “Estonian Language Technology 2018–2027”.* Estonia: Ministry of Education and Research of the Republic of Estonia.

[B46] Mohr S. (2022). Investigating English in multilingual contexts online: Identity construction in geotagged Instagram data. *Front. Commun.* 6:778050. 10.3389/fcomm.2021.778050

[B47] Mossberger K. Tolbert C. J. (2021). Digital citizenship and digital communities: How technology matters for individuals and communities. *Intern. J. E-Plann. Res.* 10 19–30. 10.4018/IJEPR.20210701.oa2

[B48] Mossberger K. Tolbert C. J. McNeal R. S. (2008). *Digital Citizenship: The Internet, Society, and Participation.* Cambridge, MA: MIT Press.

[B49] Mpofu P. Fadipe I. A. Tshabangu T. (2023). “Appreciating Indigenous African Language Media’ Practices and Processes: A Transdisciplinary Approach,” in *Indigenous African Language Media*, eds Mpofu P. Fadipe I. A. Tshabangu T. (Singapore: Palgrave Macmillan), 10.1007/978-981-99-0305-4_1

[B50] Mühlhäusler P. (1996). *Linguistic Ecology: Language Change and Linguistic Imperialism in the Pacific Region.* Milton Park: Routledge.

[B51] National Bureau of Translations (n.d.). *New Humanitarian Knowledge: 100 New Textbooks in the Kazakh Language’.* Available online at: https://100oqulyq.kz/kz.

[B52] Nelde P. H. (1987). Language contact means language conflict. *J. Multiling. Multicult. Dev.* 8 33–42. 10.1080/01434632.1987.9994273

[B53] Nordregio (2024). *National Digital Inclusion Initiatives in the Nordic and Baltic Countries. Nordregio Report 2024:3.* Stockholm: Nordregio.

[B54] OECD (2025). *Government at a Glance 2025.* Paris: OECD.

[B55] OECD (2026). *Policy Initiative: Estonian Language Technology 2018 – 2027. OECD Science, Technology and Innovation Policy (STIP) Compass Interactive Dashboard.* Paris: OECD.

[B56] OpenU (2026). *Open University of Kazakhstan [Educational platform].* Available online at: https://openu.kz/kz (accessed January 15, 2026).

[B57] 100 oqulyq (2026). *100 oqulyq [100 textbooks].* Available online at: https://100oqulyq.kz/kz (accessed January 15, 2026).

[B58] Pavlenko A. (2008). Multilingualism in post-Soviet countries: Language revival, language removal, and socialistic theîry. *Intern. J. Biling. Educ. Biling.* 11 440–475. 10.1080/13670050802271517

[B59] Petrovica S. Anohina-Naumeca A. Avanesova J. (2022). The impact of digitalisation of higher education: The case of latvia and nordic-baltic region. *Appl. Comp. Syst.* 27 19–29. 10.2478/acss-2022-0003

[B60] QazCorpus (2026). *National Corpus of the Kazakh Language. Ahmet Baitursynuly Institute of Linguistics.* Almaty: QazCorpus.

[B61] Rehm G. Marheinecke K. Hegele S. Piperidis S. Bontcheva K. Hajiè J. et al. (2020). “The European Language Technology Landschape in 2020,” in *Proceedings of the 12th International Conference on Language Resources and Evaluation (LREC 2020)*, (Marseille).

[B62] Results of the Population Census (1990). *State Committee of the Kazakh SSR for Statistics. Results of the 1989 All-Union Population Census in the Kazakh SSR: National Composition of the Population.* Moscow: Republican Information and Publishing Center.

[B63] Sarsenbieva N. F. Myrzahmetova B. S. Adylbekova J. T. (2021). *Digitalization of education in the Republic of Kazakhstan. Mir pedagogiki I psihologii: Mezhdunarodnyj nauchno-prakticheskij zhurnal, 01(54.* Available online at: https://scipress.ru/pedagogy/articles/tsifrovizatsiya-obrazovniya-v-respublike-kazakhstan.html (accessed December 25, 2025).

[B64] Schiffman H. (1996). *Linguistic Culture and Language Policy*, 1st Edn. Milton Park: Routledge, 10.4324/9780203021569

[B65] Serikbaeva A. D. (2023). Sociolinguistic features of the official language in the process of digitalization in Kazakhstan. *Tiltanym* 91 184–191. 10.55491/2411-6076-2023-3-184-191

[B66] Serikbaeva A. Olach Z. (2025). Kazakh language in the context of globalization and digital transformation: Language changes. *Tiltanym* 97 151–160. 10.55491/2411-6076-2025-1-151-160

[B67] SHR Monitor (2026). *Alienating Russian Speakers in Estonia could have Destabilizing Effects.* Netherlands: SHR Monitor.

[B68] Smagulova J. (2008). Language policies of kazakhization and their influence on language attitudes and use. *Intern. J. Biling. Educ. Biling.* 11 440–475. 10.1080/13670050802148798

[B69] Smit A. Swart J. Broersma M. (2025). Understanding digital citizenship in everyday life: Tensions between digital inclusion policies and disadvantaged citizens’ experiences. *Inform. Commun. Soc.* 29, 2450–2468. 10.1080/1369118X.2025.2571396

[B70] Spolsky B. (2004). *Language Policy.* Cambridge: Cambridge University Press.

[B71] State Programme for the Development and Functioning of Languages in the Republic of Kazakhstan for 2011–2019, (2011). *Approved by Decree of the President of the Republic of Kazakhstan dated June 29, 2011, No. 110.* Available online at: https://adilet.zan.kz/rus/docs/U1100000110 (accessed January 24, 2026).

[B72] State Programme for the Implementation of Language Policy in the Republic of Kazakhstan for 2020–2025, (2019). *Approved by the Decree of the Government of the Republic of Kazakhstan dated December 31, 2019, No. 1045.* Available online at: https://adilet.zan.kz/rus/docs/P1900001045 (accessed January 24, 2026) .

[B73] Statistics Estonia (2026). *Eesti elanikud räägivad 231 eri emakeelt [Residents of Estonia speak 231 different mother tongues].* Estonia: Statistics Estonia.

[B74] Suleimenova E. D. (2020). Globalizacija i jazykovaja politika [Globalization and language policy]. *Polilingvalnost Trans. Praktiki* 17 112–120. 10.22363/2618-897X-2020-17-1-112-120

[B75] Tlepbergen D. Akzhigitova A. Zabrodskaja A. (2023). Bottom-Up approach to language policy and planning in kazakhstan. *Societies* 13:43. 10.3390/soc13020043

[B76] Tlepbergen D. Akzhigitova A. Zabrodskaja A. (2025). Kazakh–English bilingualism in kazakhstan: Public attitudes and language practices. *Languages* 10:102. 10.3390/languages10050102

[B77] Togmanov M. Mukhituly N. Turmakhan D. Mansurov J. Goloburda M. Sakip A. et al. (2025). KazMMLU: Evaluating language models on kazakh, russian, and regional knowledge of kazakhstan. *arXiv* [Preprint] 10.48550/arXiv.2502.12829

[B78] Tripathi A. K. (2025). “Challenges of Language Survival in Digital Perspectives: Case and Context of India,” in *The Routledge Handbook of Endangered and Minority Languages*, eds Wei W. Schnell J. (Milton Park: Routledge), 171–187.

[B79] UNESCO (2021). *UNESCO Recommendations on Open Science.* Paris: UNESCO.

[B80] UNESCO Office in Almaty (2021). *Promoting Technology-Enabled Education and Skills Development in Rural and Remote Areas of Central Asia: Issue Note.* Paris: United Nations Educational, Scientific and Cultural Organization.

[B81] van Dijk T. A. (1993). Principles of critical discourse analysis. *Discourse Soc.* 4 249–283. 10.1177/0957926593004002006

[B82] Wikidata (2026). *Kazakh Wikipedia [Data set].* San Francisco, CA: Wikidata.

[B83] Wikimedia Foundation (2026). *Kazakh Wikipedia [Qazaqsha Wikipedia].* San Francisco, CA: Wikimedia Foundation.

[B84] Wodak R. Meyer M. (eds) (2015). *Methods of Critical Discourse Studies*, 3rd Edn. Thousand Oaks, CA: Sage.

[B85] Yedgina G. Dzhumabekov D. Zuyeva L. Dosova B. Kozina V. (2023). Language policy in kazakhstan in the context of world practice. *J. National. Mem. Lang. Polit.* 17 77–96. 10.2478/jnmlp-2023-0004

[B86] Yessenbekova U. M. Syzdykova A. A. Sak K. O. (2025). Zhastardyń qazaq tilin jańa media ortada qoldanu perspektivalaryn bolzhau [Forecasting the prospects for the use of the Kazakh language by young people in the new media environment]. *Bull. L.N. Gumilyov Eur. Natl. Univ. J. Series* 152 34–65. 10.32523/2616-7174-2025-152-3-34-65

[B87] Zaugg I. A. Hossain A. Molloy B. (2022). Digitally-disadvantaged languages. *Internet Policy Rev.* 11 1-11. 10.14763/2022.2.1654

[B88] Zharkynbekova S. Ayupova G. Galiyeva B. Shakhputova Z. Zabrodskaja A. (2025a). Ethno-Linguistic identity of kazakhstani student youth in modern multinational context of kazakhstan (Sociolinguistic Analysis of Empirical Research). *Languages* 10 33. 10.3390/languages10020033

[B89] Zharkynbekova S. Shakhputova Z. Galiyeva B. Absadyk A. (2025b). Value priorities of student youth in the multi-ethnic space of kazakhstan and their influence on intercultural communications. *J. Media* 6:32. 10.3390/journalmedia6010032

[B90] Zipf G. K. (1949). *Human Behaviour and the Principle of Least Effort.* Cambridge, MA: Harvard University Press.

